# Analysis of Biomarkers of Oxidative Stress and Inflammation in Mexican Patients With Type 2 Diabetes and Chronic Kidney Disease

**DOI:** 10.7759/cureus.112796

**Published:** 2026-07-16

**Authors:** Francisco Mendoza-Carrera, Gloria Elizabeth Vazquez-Rivera, Renato Parra-Michel, Rosalba Orozco-Sandoval, Luis Alberto Salazar-Soltero, Lourdes del Carmen Rizo-de la Torre, Caridad Aurea Leal-Cortes

**Affiliations:** 1 Molecular Medicine Division, Centro de Investigación Biomédica de Occidente, Instituto Mexicano del Seguro Social, Guadalajara, MEX; 2 Nephrology Department, Hospital General Regional No. 46, Instituto Mexicano del Seguro Social, Guadalajara, MEX; 3 Clinical Coordination of Education and Health Research, Unidad de Medicina Familiar No. 3, Instituto Mexicano del Seguro Social, Guadalajara, MEX; 4 Surgical Research Division, Centro de Investigación Biomédica de Occidente, Instituto Mexicano del Seguro Social, Guadalajara, MEX

**Keywords:** biomarkers, cytokines, diabetic kidney disease, inflammation, oxidative stress

## Abstract

Background: Oxidative stress (OS) and persistent low-grade inflammation are frequently observed in hyperglycemic conditions such as diabetes. The persistent hyperglycemic environment in patients with diabetes stimulates the production of advanced glycation end-products and reactive oxygen species, which could disturb the redox balance and lead to OS. In turn, OS can dysregulate the inflammatory response and promote tissue damage, leading to common complications in diabetes. This study aims to evaluate OS and proinflammatory cytokines in adult patients with diabetes and chronic kidney disease (CKD).

Objective: This study aimed to analyze the levels of OS and inflammation biomarkers in Mexican patients with type 2 diabetes (T2D) and CKD.

Methods: Serum levels of superoxide dismutase (SOD), malondialdehyde (MDA), interleukin (IL)-1β, IL-6, and tumor necrosis factor (TNF)-α were determined using Luminex and enzyme-linked immunosorbent assay in a sample of 147 Mexican patients with T2D. Patients were divided into two groups: those with CKD (n = 81) and those without CKD (n = 66). Levels of analyzed biomarkers were compared between the groups. Correlations and multivariate linear regression analyses were performed.

Results: Chronicity of disease, systolic blood pressure (SBP), uric acid levels, and prevalence of hypertension and hyperuricemia were higher in patients with CKD. SODs levels were higher in patients with CKD, whereas no difference in MDA concentrations was observed. IL-6 and TNF-α were significantly higher in patients with CKD. SOD levels positively correlated with SBP, uric acid, and TNF-α but negatively with glycated hemoglobin and estimated glomerular filtration rate (eGFR). Besides the major factors associated with CKD, such as duration of diabetes, hyperuricemia, and high blood pressure, elevated levels of IL-1β, IL-6, TNF-α, and SOD were also associated. Of the biomarkers analyzed, SOD and TNF-α were strong predictors of eGFR decline in patients with diabetes.

Conclusion: Serum concentrations of SOD and the proinflammatory cytokines IL-1β, IL-6, and TNF-α were increased in patients with T2D and CKD and could be considered good biomarkers of OS and inflammation status. Monitoring of these molecules could contribute to better management of patients with T2D.

## Introduction

Type 2 diabetes (T2D) accounts for nearly 90% of the estimated 537 million cases of diabetes globally [1]. In Mexico, according to the most recent National Survey of Health and Nutrition, the national prevalence of T2D was 17%. Of this total, 11.6% comprised individuals with a diagnosed case of T2D, while 5.4% represented individuals who had not received a diagnosis [2]. Also, it has been reported that up to 40%-50% of the Mexican T2D patients had chronic kidney disease (CKD), and nearly 60% of patients in replacement therapy of the kidney function had diabetes as a cause of end-stage kidney disease [3,4]. Given that T2D significantly impacts the health and socioeconomic status of both patients and national health systems, understanding its pathogenesis and how to prevent its complications is a major goal of intensive research. It is well documented that the persistent hyperglycemic environment in patients with diabetes stimulates the production of advanced glycation end-products and reactive oxygen species (ROS), which, if not regulated, might cause endothelial cell injury and vascular disease [5,6]. Additionally, other components of the metabolic syndrome, such as increased lipid levels, as well as obesity and high blood pressure, are frequently found in patients with diabetes, all of which are ROS-generating conditions [7]. As a defense, cells can mitigate the harmful effects of ROS by upregulating genes coding for antioxidant proteins, such as superoxide dismutase (SOD), glutathione peroxidase (GPx), and catalase [6,8]. When the antioxidant response is exceeded by oxidant molecules, an oxidative stress (OS) state is established, which can stimulate the production of proinflammatory cytokines and may result in cell or tissue damage [9,10]. OS is linked to various metabolic pathways that exacerbate insulin resistance and inflammation, generating a self-perpetuating cycle of damage that leads to endothelial damage, the hallmark of the major complications occurring in patients living with diabetes [6,11,12].

The study of OS biomarkers in diabetes is crucial for understanding the pathophysiology of diabetes and its complications, as they reflect the balance between OS and the body's antioxidant defenses. Among ROS biomarkers, malondialdehyde (MDA), a product of lipid peroxidation, and SOD enzyme activity are indicators of oxidative status and antioxidant system response, respectively, in experimental and clinical settings [13-15]. On its part, inflammation status can be evaluated by the measurement of key mediators of the inflammatory response such as tumor necrosis factor (TNF)-α, interleukin (IL)-6, and C-reactive protein [14]. It has been suggested that monitoring of both ROS and inflammation markers can provide insights into the progression and management of diabetes [6,14]. However, controversy over which biomarkers should be selected for a proper OS assessment remains without consensus, and more biomarkers are under biomedical research [6,8]. In addition, the use of antioxidants has been proposed as an adjuvant in the clinical management of patients with diabetes to improve their response to conventional therapy and to reduce the risk of major complications at renal and cardiovascular levels [5].

It is well-known that inflammation and OS are concurrently present in diabetes and CKD. Studies assessing differences in OS-related molecules and certain pro-inflammatory cytokines in Mexican patients with T2D by the presence of CKD are limited [16-18]. Therefore, this study aimed to determine the levels of two main OS biomarkers, MDA and SOD, and three proinflammatory cytokines, IL-1β, IL-6, and TNF-α, in a sample of Mexican patients with T2D and to compare them by the presence of kidney disease.

## Materials and methods

Patient recruitment

A cross-sectional analytical study was carried out including 147 patients with a diagnosis of T2D according to the American Diabetes Association, defined as an occasional fasting glucose ≥126 mg/dL or an altered glucose tolerance test, or continuous use of antidiabetic medication [19]. The primary criterion for patient enrollment was being diagnosed with T2D for at least one year. The patients are under medical follow-up due to their diabetic condition and were selected according to the presence or absence of kidney disease. Those with CKD (n = 81) were recruited from the Nephrology Department of the Regional General Hospital No. 46 of the Mexican Institute for Social Security (IMSS) in Guadalajara, Jalisco, Mexico, between January 2021 and December 2022. Participants without CKD (n = 66) were enrolled from a primary care facility, specifically the Family Medicine Unit No. 3. Both the hospital and the Family Medicine Unit belong to IMSS. Recruitment was conducted in the same geographic location and during the same period as for those with CKD. The presence of kidney disease was considered if the estimated glomerular filtration rate (eGFR) was below 60 mL/minute/1.73 m^2^ according to the Kidney Disease: Improving Global Outcomes guidelines [20]. All procedures adhered to international regulations governing research involving human subjects, including the Declaration of Helsinki and the Council for International Organizations of Medical Sciences. The study was approved by the Ethics Research Committee of the Mexican Social Security Institute (approval number: R-2021-1305-007). All patients signed a letter of informed consent before their enrollment in the study.

Body mass index was determined by dividing body weight (in kilograms) by the square of the height (in meters); blood pressure was measured from the arm’s middle portion using a mercury sphygmomanometer after 10 minutes of rest in the physician's office. Presence of hypertension was considered if diastolic or systolic blood pressures (SBPs) were ≥140 and/or ≥90 mmHg [21], use of antihypertensive medication, or previous diagnosis of hypertension was established. Peripheral blood samples were collected by venipuncture into sterile vacuum tubes. For serum separation, samples were left to clot for at least 30 minutes before centrifugation at 4°C for 20 minutes. Then, the obtained serum was aliquoted and sent for measurement of biochemical parameters or stored at -80°C until analysis. Biochemical parameters, including glucose, total cholesterol, triglycerides, glycated hemoglobin, and uric acid, were measured using an automated analyzer (Vitros 300 Integrated System, Ortho Clinical Diagnostics, Raritan, NJ) according to the manufacturer’s instructions. Serum levels of MDA were determined by using a thiobarbituric acid-based colorimetric assay kit (Cayman Chemical, Ann Arbor, MI), while SOD levels were measured by using an enzyme-linked immunosorbent assay kit (Mybiosource, San Diego, CA) following the manufacturer’s recommendations. Cytokines IL-1β, IL-6, and TNF-α were determined by an immunofluorescence magnetic bead-coupled multiplex kit (Human Bone Magnetic Bead Panel-Bone Metabolism Multiplex Assay, Merck Millipore, Burlington, MA) read on a Luminex 200 instrument (Luminex Corporation, Austin, TX).

Statistical analysis

Categorical data are presented as counts and percentages, whereas continuous variables are presented as mean ± standard deviation or median interquartile 25th-75th range according to the assessment of normality or nonnormality of data by using the Kolmogorov-Smirnov test. The chi-square test was used to compare categorical variables, while Student's t-test or Mann-Whitney U test was used to compare means or medians between groups, as appropriate. Bivariate (two-tailed Pearson test) and multivariate analyses (multiple linear regression models) were used to analyze our population data. Backward models were used to evaluate the relationships between eGFR and both OS and inflammatory biomarkers, as well as other relevant variables. Only those parameters with statistical significance in the bivariate correlation analysis were included in the models. Analyses were conducted using the Statistical Package for the Social Sciences, version 24 (IBM Corp., Armonk, NY), for Mac, with statistical significance defined as a p value less than 0.05 (p < 0.05) for all tests.

## Results

The comparison of sociodemographic and clinical variables by kidney disease status is shown in Table 1. There were no differences in age or sex between the groups. Compared with patients without CKD, time with diabetes, SBP, uric acid levels, and prevalence of hypertension and hyperuricemia were higher in patients with CKD.

**Table 1:** Patient characteristics based on the presence of kidney disease Categorical variables are presented as counts and percentages. Numerical variables are presented as mean ± standard deviation or median (interquartile range) according to the normality of the distribution (see the Materials and Methods section). Chi-square test was performed to compare categorical variables, and Student's t-test or Mann-Whitney's U test was performed for numerical variables according to normality or nonnormality of distribution, respectively CKD: diabetic kidney disease; eGFR: estimated glomerular filtration rate

Variable	CKD (n = 81)	Non CKD (n = 66)	Statistic	p
Males, n (%)	46 (57)	35 (53)	Χ^2^ = 0.208	0.7387
Age (years)	66 ± 11	62 ± 11	t = -1.748	0.0825
Body mass index (kg/m^2^)	28.6 ± 4.9	29.5 ± 4.9	t = 1.035	0.3023
Systolic blood pressure (mmHg)	143 (126-165)	120 (120-131)	U = 3545.500	<0.0001
Diastolic blood pressure (mmHg)	80 (73-87)	80 (74-80)	U = 2654.500	0.1215
Duration of diabetes (years)	16.4 ± 9.6	11.2 ± 8.9	t = -3.098	0.0022
Fasting glucose (mg/dL)	119 (101-163)	135 (102-178)	U = 2258.500	0.1059
Glycated hemoglobin (%)	8.0 ± 2.3	8.3 ± 2.1	t = 0.576	0.5311
Uric acid (mg/dL)	6.9 ± 1.8	5.3 ± 1.4	t = -5.954	<0.0001
Total cholesterol (mg/dL)	184 ± 62	199 ± 45	t = 1.606	0.1112
Triglycerides (mg/dL)	214 ± 135	174 ± 61	t = -2.326	0.02188
eGFR (mL/min/1.73 m^2^)	37 ± 12	90 ± 17	t = 21.66	<0.0001

Values of OS and inflammation biomarkers by the presence of CKD are given in Table 2. Regarding OS markers, SOD levels were higher in patients with CKD (2.0 vs. 1.4, p < 0.001), whereas MDA concentrations showed no difference. Regarding cytokines, TNF-α was significantly higher in patients with CKD (4.1 vs. 1.6 (p < 0.001). Similarly, IL-1β and IL-6 were shown to be slightly but significantly increased in CKD patients (0.25 vs. 0.15, p = 0.015 and 0.25 vs. 0.20, p <0.001, respectively). Additionally, the results of correlation analyses between concentrations of MDA and SODs with clinical, biochemical, and inflammatory parameters are presented in Table 3. SOD level correlated positively with SBP (r = 0.293, p = 0.001), uric acid (r = 0.225, p = 0.007), triglycerides (r = 0.168, p = 0.049), and TNF-α (r = 0.351, p = 0.001), and negatively with eGFR (r = -0.463, p <0.001). There were no significant correlations between MDA and any of the evaluated parameters.

**Table 2:** Oxidative stress and inflammatory biomarkers patients with type 2 diabetes based on the presence of chronic kidney disease Numerical data are presented as mean ± standard deviation or median (interquartile range) according to the normality of the distribution (see the Materials and Methods section). Comparisons were made using Student’s t-test or Mann-Whitney’s U test, as appropriate CKD: chronic kidney disease; MDA: malondialdehyde; SOD: superoxide dismutase; IL: interleukin; TNF: tumor necrosis factor

Variable	CKD (n = 81)	Non-CKD (n = 66)	Statistic	p
MDA (ng/mL)	2.70 ± 2.30	2.81 ± 2.01	t = 0.501	0.617
SOD (ng/mL)	2.04 ± 0.72	1.41 ± 0.61	t = -5.545	<0.0001
IL-1β (pg/mL)	0.25 (0.23-0.34)	0.15 (0.15-0.25)	U = 2,236.000	0.0152
IL-6 (pg/mL)	0.25 (0.25-1.00)	0.20 (0.15-0.25)	U = 2,449.000	<0.0001
TNF-α (pg/mL)	4.10 ± 2.20	1.60 ± 1.60	t = -7.240	<0.0001

**Table 3:** Correlations of concentrations of MDA and SODs with anthropometrical, biochemical, and inflammatory parameters in patients with type 2 diabetes SOD: superoxide dismutase; MDA: malondialdehyde; eGFR: estimated glomerular filtration rate; IL: interleukin; TNF: tumor necrosis factor

Variable	SOD		MDA	
	Pearson r	p	Pearson r	p
Age (years)	-0.032	0.701	-0.006	0.94
Body mass index (kg/m^2^)	-0.089	0.294	0.161	0.058
Systolic blood pressure (mmHg)	0.293	0.001	-0.036	0.677
Diastolic blood pressure (mmHg)	0.121	0.163	-0.141	0.104
Uric acid (mg/dL)	0.225	0.007	-0.035	0.681
Glucose (mg/dL)	-0.070	0.404	0.129	0.123
Glycated hemoglobin (%)	-0.189	0.057	0.015	0.884
Total cholesterol (mg/dL)	-0.069	0.419	-0.042	0.621
Triglycerides (mg/dL)	0.168	0.049	-0.105	0.223
eGFR (mL/min/1.73 m^2^)	-0.463	<0.001	0.086	0.307
IL-1β (pg/mL)	0.111	0.226	-0.104	0.262
IL-6 (pg/mL)	0.185	0.043	-0.136	0.14
TNF-α (pg/mL)	0.351	0.001	-0.073	0.431
SOD (ng/mL)	-	-	-0.060	0.475
MDA (ng/mL)	-0.060	0.475	-	-

Multiple linear models were employed to evaluate factors that affect renal function in both the overall patient cohort and subgroups defined by the presence of CKD (Table 4). Only variables with significant correlations in previous bivariate analyses were included, with eGFR as the dependent variable. In the entire patient population, age (B = -0.960; 95% confidence interval (CI): -1.361, -0.559), uric acid (B = -4.886; 95% CI: -7.908, -1.864), and TNF-α (B = -5.613; 95% CI: -7.772, -3.455) may have a negative impact on eGFR. Regarding patients without CKD, age (B = -0.637; 95% CI: -1.043, -0.232), triglycerides (B = -0.124; 95% CI: -0.201, -0.047), IL-6 (B = -1.240; 95% CI: -2.099, -0.243), and SOD (B = -14.602; 95% CI: -23.612, -5.591) were significantly associated with renal function, whereas TNF-α was the sole factor associated with reduced eGFR among patients with CKD (B = -1.788; 95% CI: -3.450, -0.127).

**Table 4:** Predictors of renal function change in the entire sample and stratified according to the presence of CKD R2 of the models: ^a^0.574, ^b^0.571, and ^c^0.075. Variance inflation factors were calculated to avoid collinearity, and the maximum value was 2.5 for IL-6 with TNF-α. The remaining parameters for all the tested models are available upon request eGFR: estimated glomerular filtration rate; IL: interleukin; SOD: superoxide dismutase; TNF-α: tumor necrosis factor-alpha; CKD: chronic kidney disease

Parameter	B (95% confidence interval)	p
eGFR (whole sample)^a^		
Age (years)	-0.960 (-1.361, -0.559)	<0.0001
Uric acid (mg/dL)	-4.886 (-7.908, -1.864)	0.0021
Triglycerides (mg/dL)	-0.051 (-0.091, -0.011)	0.0141
TNF-α (pg/mL)	-5.613 (-7.772, -3.455)	<0.0001
SOD (ng/mL)	-6.404 (-13.302, 0.494)	0.0685
eGFR (T2D without CKD)^b^		
Age (years)	-0.637 (-1.043, -0.232)	0.0032
Triglycerides (mg/dL)	-0.124 (-0.201, -0.047)	0.0031
IL-6 (pg/mL)	-1.240 (-2.099, -0.243)	0.0056
SOD (ng/mL)	-14.602 (-23.612, -5.591)	0.0033
eGFR (T2D with CKD)^c^		
TNF-α (pg/mL)	-1.788 (-3.450, -0.127)	0.0350

## Discussion

In this study, markers of OS and inflammation were analyzed in a sample of Mexican patients with T2D with and without kidney disease. Comparisons of variables were conducted based on the presence or absence of kidney disease. The most notable finding was the association with traditional risk factors such as disease chronicity, high blood pressure, and hyperuricemia. Also, levels of the antioxidant enzyme SOD and inflammatory cytokines were associated with diabetic nephropathy. SOD concentration was negatively correlated with renal function (r = -0.463, p < 0.001). In contrast, MDA levels were similarly distributed between the groups. These findings are noteworthy because patients with diabetes exhibit a redox imbalance toward ROS generation, which, if not controlled, can lead to OS [7]. Notably, increased levels of SOD, an antioxidant enzyme, have been observed in patients with kidney disease. To mitigate the potential oxidative damage caused by ROS, the organism initiates the synthesis of both protein and nonprotein compounds capable of neutralizing the effects of ROS. One of these molecules is SOD, which catalyzes the dismutation of superoxide (O_2_^•-^), generating hydrogen peroxide, which in turn is converted into water by the action of catalase, GPx, and peroxidases [22]. Several studies have reported decreased SOD levels in several metabolic conditions, including diabetes and kidney disease [6,14,18]. Conversely, it has also been observed that higher serum and urinary SOD levels are found in patients with diabetes and CKD compared with those patients without CKD [23]. Additionally, exposure to an SOD isoform was associated with improved antioxidant response and kidney function in a murine model of nephropathy and albuminuria [23]. There are at least three known SODs isoforms (SOD1-3) in mammals with specific expression and action patterns [22,24], of which only SOD3 can be secreted into the extracellular space in most peripheral tissues and exported through circulation, exerting its protective role in distant organs [22]. Since only serum samples were included in our study, the elevated SOD levels observed in patients with CKD are attributable to the SOD3 isoform and may indicate the body's systemic response to OS induced by factors such as hyperglycemia or renal impairment. However, no differences in SOD levels among CKD stages were observed (see the Appendix). Nevertheless, our study did not demonstrate differences in SOD levels across CKD stages, which may be attributable to the limited sample size. Also, other key participants in the antioxidant response, besides SOD, such as GPx, glutathione S-transferases, and catalase, which were not measured in this study, could be simultaneously operating to restore redox balance. Future studies quantifying specific isoforms of SOD or other antioxidant enzymes at both the systemic and kidney levels will help to better elucidate the involvement of protective oxidation mechanisms in patients with diabetes and kidney disease. On the other hand, MDA concentrations were not associated with eGFR or the presence of CKD, which differs from previous studies reporting elevated levels of this molecule in patients with diabetes who have complications such as CKD [5,6]. Because MDA is a product of lipid peroxidation, the absence of significant differences in this OS biomarker between the study groups may be attributable to comparable lipid concentrations among patients with and without kidney disease. Alternatively, MDA quantification may not be the best reflection of the oxidation status of patients with T2D, despite being a widely used OS marker, perhaps due to the technical convenience of its measurement. However, more specific markers in diabetes and CKD, such as the nucleotide-derived 8-hydroxyguanosine and methylglyoxal, among others, and recently proposed markers such as S100A9 and Selectin L [8], could contribute to a better description of the OS status in these patients.

It is well known that inflammation and OS are concurrently present in diabetes and CKD, and it has been suggested that monitoring both ROS and inflammatory markers can provide insights into the progression and management of diabetes [6,14]. In our study, the levels of the three inflammatory markers, IL-1β, IL-6, and TNF-α, were elevated in patients. Additionally, TNF-α concentrations were strongly and negatively correlated with eGFR and CKD stages (see the Appendix). It has been suggested that elevated concentrations of inflammatory mediators may contribute to endothelial injury and influence the progression of vascular complications, including nephropathy. IL-6 and TNF-α can directly induce apoptosis in renal tubular epithelial cells through various signaling pathways and serve as independent risk factors for poor prognosis in patients with CKD [14]. On its part, IL-1β plays an important role in the inflammasome activation, which, in turn, induces profibrotic pathways [24,25]. With respect to OS markers, only SOD levels demonstrated an association with CKD, exhibiting a negative correlation with eGFR and a positive correlation with TNF-α. These findings are consistent with previous studies that highlight a strong relationship among OS, inflammation, and renal function [14,15,26].

Another factor strongly associated with renal function was uric acid. This metabolite was found to be significantly correlated with SOD and cytokine levels. On its own, high uric acid levels have been associated with increases in inflammatory markers, perhaps due to the formation of microcrystals in the intercellular space, which can directly induce inflammation as well as endothelial and increased vascular risk [26]. Alternatively, extracellular uric acid can exert antioxidant activity, which could be considered a defense or protective mechanism against systemic OS, of what happens at the renal level [27]. Unfortunately, these markers were measured only in serum, which may reflect a different environment than that at the kidney level.

Limitations

Some limitations of our study must be recognized. First, the cross-sectional design permitted only a single measurement, which limited our ability to obtain a comprehensive understanding of patients' overall condition. Second, information on medications, dietary intake, or food supplement use that might affect patients' OS status was not considered. Finally, the sample size, although adequate for detecting initial associations, may explain the weak associations observed, which forces us to interpret our results with caution. Nevertheless, our research offers pertinent data on the combined assessment of OS and inflammatory biomarkers in individuals with T2D and kidney disease. Finally, the results of this study may guide future research efforts focused on improving clinical outcomes for people with diabetes, particularly by prioritizing the prevention or reduction of serious complications associated with the condition. Further studies, whether observational or interventional, involving larger patient cohorts, are required to enhance our understanding of the combined effects of OS and inflammation in diabetes, particularly their influence on kidney function.

## Conclusions

Our study revealed that serum levels of SOD and the proinflammatory cytokines IL-1β, IL-6, and TNF-α are increased in patients with T2D and kidney disease. Metabolic markers, including uric acid, demonstrated associations with both SOD and cytokines and could serve as reliable predictors of declining kidney function. Implementing strategies focused on careful monitoring of redox balance and inflammatory responses may help reduce or prevent major complications in individuals with diabetes.

## Appendices

**Table 5:** Bivariate correlations of clinical, metabolic, biochemical, and inflammatory markers Data are presented as Pearson’s correlation coefficients (above the diagonal) and p values (below the diagonal) SBP: systolic blood pressure; DBP: diastolic blood pressure; BMI: body mass index; UA: uric acid; HbA1c: glycated hemoglobin; t-Chol: total cholesterol; TG: triglycerides; eGFR: estimated glomerular function rate; IL: interleukin; TNF-α: tumor necrosis factor-alpha; SOD: superoxide dismutase; MDA: malondialdehyde

	Age	SBP	DBP	BMI	UA	Glucose	HbA1c	t-Chol	TG	eGFR	IL-1β	IL-6	TNF-α	SOD	MDA
Age	1	0.236	-0.056	-0.162	-0.044	-0.022	0.023	-0.252	-0.346	-0.223	-0.107	-0.007	0.058	-0.032	-0.006
SBP	0.005	1	0.547	-0.050	0.205	-0.163	-0.073	0.068	-0.002	-0.422	-0.127	0.026	0.439	0.293	-0.036
DBP	0.512	<0.001	1	-0.014	0.115	-0.109	-0.100	0.082	0.079	-0.063	-0.193	0.129	0.082	0.121	-0.141
BMI	0.054	0.566	0.868	1	0.008	-0.124	0.072	0.2	0.078	0.106	-0.007	-0.099	0.01	-0.089	0.161
UA	0.598	0.018	0.186	0.93	1	-0.152	0.009	-0.086	0.147	-0.460	0.092	0.059	0.426	0.225	-0.035
Glucose	0.791	0.057	0.203	0.141	0.071	1	0.191	-0.019	0.162	0.177	-0.055	-0.122	-0.085	-0.070	0.129
HbA1c	0.82	0.47	0.321	0.475	0.923	0.1991	1	0.121	0.113	0.104	-0.028	0.041	0.0141	-0.189	0.015
t-Chol	0.002	0.437	0.348	0.019	0.314	0.82	0.228	1	0.405	0.114	-0.052	-0.026	-0.074	-0.069	-0.042
TG	<0.001	0.982	0.368	0.371	0.088	0.056	0.268	<0.001	1	-0.192	0.123	0.036	0.162	0.168	-0.105
eGFR	0.007	<0.001	0.463	0.207	<0.001	0.032	0.295	0.177	0.023	1	-0.075	-0.033	-0.599	-0.463	0.086
IL-1β	0.241	0.18	0.041	0.936	0.325	0.552	0.806	0.58	0.19	0.414	1	0.434	0.175	0.111	-0.104
IL-6	0.942	0.789	0.175	0.29	0.529	0.184	0.713	0.781	0.705	0.722	<0.001	1	-0.033	0.185	-0.136
TNF-α	0.528	<0.001	0.392	0.998	<0.001	0.355	0.306	0.425	0.084	<0.001	0.055	0.721	1	0.351	-0.073
SOD	0.7	0.001	0.163	0.294	0.007	0.4	0.057	0.419	0.049	<0.001	0.226	0.043	0.001	1	-0.060
MDA	0.94	0.677	0.104	0.058	0.681	0.123	0.884	0.621	0.223	0.307	0.262	0.14	0.431	0.475	1
